# Effect of the knee position during wound closure after total knee arthroplasty on early knee function recovery

**DOI:** 10.1186/s13018-014-0079-2

**Published:** 2014-08-23

**Authors:** Siqun Wang, Jun Xia, Yibin Wei, Jianguo Wu, Gangyong Huang

**Affiliations:** 1 Department of Orthopaedics, Huashan Hospital Affiliated to Fudan University, Shanghai 200040, China

**Keywords:** Anterior knee pain, Range of motion, Wound closure, Total knee arthroplasty

## Abstract

**Objective:**

This study investigated the effect of the knee position during wound closure on early knee function recovery after total knee arthroplasty (TKA).

**Methods:**

This study included 80 primary total knee arthroplasties due to osteoarthritis. The patients were randomized according to the type of wound closure: extension group for full extension and flexion group for 90° flexion. The incision of articular capsule was marked for precise wound alignment. In the flexion group, the knee was kept in high flexion for 1 to 2 min after wound closure. The two groups were treated with the same postoperative rehabilitation exercises. The range of motion (ROM), visual analogue scale (VAS) score of anterior knee pain, Knee Society Score (KSS) and postoperative complications were assessed at 6 weeks, 3 months and 6 months, postoperatively.

**Results:**

At 6 weeks and 3 months postoperatively, the ROM in flexion group was 98.95 ± 10.33° and 110.05 ± 4.93° respectively, with 87.62 ± 8.92° and 95.62 ± 6.51° in extension group, respectively; The VAS score of anterior knee pain in flexion group was 2.02 ± 1.38 and 2.21 ± 0.87, respectively, with 2.57 ± 1.07 and 2.87 ± 0.83 in extension group, respectively. The ROM and VAS pain score of the two groups were significantly different at these two time points, with no significant difference at 6 months postoperatively. The two groups were not significantly different in KSS, and no apparent complication was observed at three time points.

**Conclusion:**

Marking the articular capsule incision, wound closure in flexion and high flexion after wound closure can effectively decrease anterior knee pain after TKA and promote the early recovery of ROM.

## Introduction

Total knee arthroplasty (TKA) has been considered a successful surgical method in the treatment of knee osteoarthritis. TKA can effectively remove pain associated with joint activities and can recover the range of motion (ROM), which is closely related to the degree of satisfaction in patients [1]. Previous studies reported that the incidence of anterior knee pain after primary TKA is from 10% to 15% [2]–[4]. The reason remains unclear. Nevertheless, this phenomenon may be associated with patient factors, degree of patellar cartilage damage, prosthesis design, patellar resurfacing, surgical technique and treatment of soft tissue around patella [5],[6]. A simple soft-tissue tension in the anterior knee after TKA can cause knee pain and loss of ROM [7]. Surgical technique significantly influences joint function recovery after TKA. Research mainly focuses on flexion and extension gap balance, rotational alignment and medial and lateral collateral ligament balance. The traditional knee wound closure in extension may lead to soft-tissue misalignment and relative shortening of the knee extension device, resulting in higher soft-tissue tension of the anterior knee in flexion. This condition may lead to anterior knee pain and may limit postoperative ROM recovery [8]. The effect of wound closure in knee flexion on ROM has been reported differently. Emerson et al. [9] indicated that wound closure in flexion contributes to ROM recovery, but Masri et al. [10] suggested otherwise. In the reported cases of wound closure, the angle of flexion did not exceed 60° and has no comprehensive treatment on soft tissue. In wound closure in extension, failure to mark the preoperative soft tissue may lead to in situ closure without complete anatomy and increased local tension of the soft tissue in knee flexion. In this study, we marked the capsular incision during the arthrotomy, sutured the wound in 90° flexion and kept the knee over-flexed for soft-tissue tension rebalance of the sutured area during wound closure. This study aims to investigate the effects of these measures on anterior knee pain and early knee function recovery.

## Materials and methods

### General data

A total of 80 patients (18 males and 62 females; 57 years old to 83 years old; average age, 68.26 ± 9.08 years; body mass index, 25.96 ± 3.65 kg/m; 80 knees) from January 2009 to August 2010 were enrolled in this study. All patients had been treated with primary TKA for osteoarthritis. The exclusion criteria were as follows: knee surgery history (femur or tibia osteotomy, knee extension device surgery and arthroscopic surgery), patella fracture history, knee valgus (>15°, requiring lateral retinacular release) or fixed-flexion deformity, knee infection history and neuromuscular disorders affecting knee motion. Stata 7.0 statistical software was used to randomly divide all patients into the extension group and flexion group, with 40 patients in each group. Age, gender, body mass index, ROM, visual analogue scale (VAS) pain score of anterior knee and Knee Society Score (KSS, American Society of Knee Surgery) were not significantly different between the two groups (Table 1). Thus, extension group and flexion group were comparable.

**Table 1:** General data in two groups (mean ± SD)

**Group**	**Age (years)**	**Gender**	**Body mass index (kg/m**^**2**^**)**	**Anterior knee VAS pain score**	**ROM (°)**	**KSS**	
						**Knee**	**Function**
Extension group (40)	67.87 ± 6.47	9/31	24.94 ± 4.64	7.97 ± 1.37	84.23 ± 3.68	46.02 ± 3.20	48.75 ± 2.03
Flexion group (40)	68.34 ± 7.09	7/33	25.00 ± 3.88	8.02 ± 1.08	82.11 ± 4.25	46.15 ± 2.78	47.37 ± 1.60
*t*	0.92	1.526	−0.07	0.63	1.69	0.07	−0.36
*P*	0.34	0.58	0.93	0.55	0.75	0.96	0.73

After the completion of evaluation according to the enrolment and exclusion criteria, an informed consent was signed by the patients. Randomisation was performed prior to surgery to determine the patients to be assigned in each group. Surgery was performed by the physicians who did not participate in the preoperative grouping and postoperative evaluation. Postoperative evaluation was conducted by the physicians who were unaware of the grouping. This study was conducted in accordance with the declaration of Helsinki. This study was conducted with approval from the Ethics Committee of Fudan University. Written informed consent was obtained from all participants.

### Surgical method

**Figure 1:**
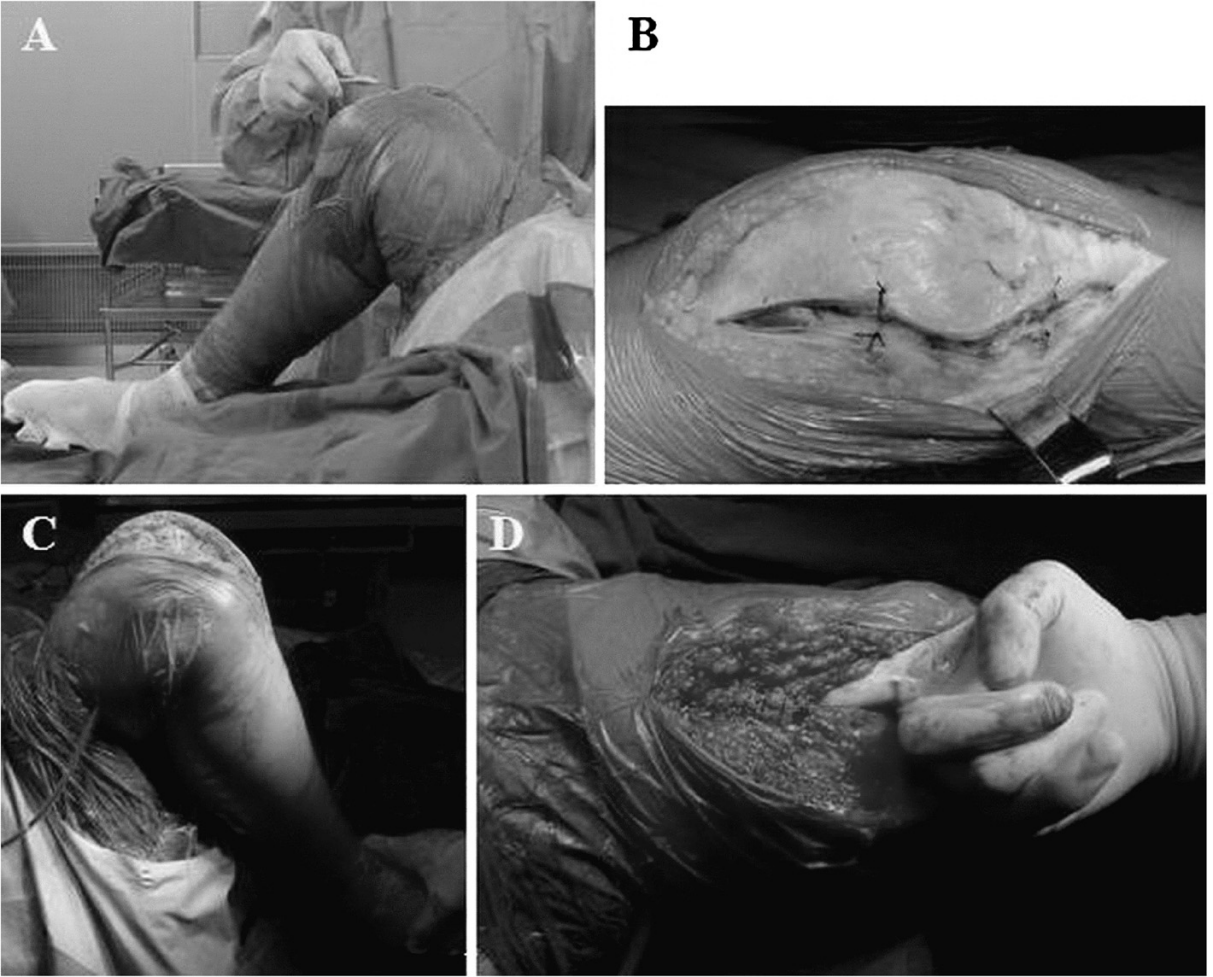
**Intra-operative technical treatment in the flexion group. (A)** Knee wound closure in 90° flexion during the TKA. **(B)** Suture mark on incision of articular capsule. **(C)** High knee flexion after wound closure. **(D)** Checking the interspace between quadriceps tendon and suprapatellar bursa after tendon closure in flexion.

All patients were administered with general anaesthesia and subjected to TKA through parapatellar medial approach by the same doctor. The skin, patellar tendon and upper and lower patella poles with articular capsule incision were marked for precise soft-tissue alignment in closure. An equivalent osteotomy was conducted on the femur and tibia, and the posterior knee osteophyte was removed. The tibia posterior slope was 3°, and Smith-Nephew Genesis II prosthesis was implanted. After osteophyte removal, the patella was shaped using an electric pendulum saw without patellar resurfacing. Prior to skin incision, a tourniquet was placed in flexion and was released after bone cement hardening. During surgery, cocktail analgesic injection [11] was injected into the articular capsule, suprapatellar bursa and infrapatellar fat pad. In the extension group, the wound closure was performed in full extension (Figure 1A). In the flexion group, during flexion the articular capsule was incised and marked by a stitch, which facilitated the accurate joint of soft tissue during suture. In the 90° flexion, the articular capsule, soft tissue and skin were enclosed. The knee was kept in high flexion for 1–2 min after wound closure to balance the uneven tension of soft tissue in the suture site (Figure 1B). All patients did not undergo patellar replacement. However, the osteophyte in all patellas was removed and subsequently underwent patellar articular surface formation via a pendulum saw. All patients went through primary TKA, and patients with excessive deformity were excluded. Lateral retinacular release was not conducted to ensure the comparability of this study. After 24 hrs, the negative pressure drainage was removed, and the patients could perform full weight-bearing walk.

### Postoperative treatment

After the surgery, the thigh of the patient was elevated to 60° (45° knee flexion) using a bracket. After 24 hrs, the negative pressure drainage was removed. The two groups were treated with the same postoperative rehabilitation exercises. At 2 days postoperatively, quadriceps contraction training was conducted with full weight-bearing walk under the help of a walking aid, which prevents falls. After 2 weeks, the knee was flexed at 90°, and the patient independently walked with weight-bearing. Corresponding corrections had been made. In the original study design, the follow-up time was 1 year. However, the two groups were not significantly different after six postoperative months.

### Efficacy observation

Efficacy of the surgery was observed using the blind method. At 6 weeks, 3 months and 6 months postoperatively, the ROM, VAS pain score of the anterior knee and KSS were assessed, as well as the knee extension lag and other complications.

### Statistical analysis

Data were expressed as mean ± standard deviation. Statistical analysis was performed using SPSS 11.5 statistical software (SPSS Inc., Chicago, IL, USA). Moreover, *t*-test and chi-square test were used to analyse the measurement data and enumeration data, respectively. *P* < 0.05 was considered statistically significant.

## Results

### ROM

At 6 weeks and 3 months postoperatively, the ROM in the flexion group was 98.95 ± 10.33° and 110.05 ± 4.93°, whereas 87.62 ± 8.92° and 95.62 ± 6.51° in the extension group, respectively. The recovery of ROM in the flexion group was significantly higher and faster than that in the extension group (*P* < 0.05). This finding indicated that the closure in flexion was beneficial to postoperative early ROM recovery. ROM was not significantly different between the two groups at 6 months postoperatively (*P* > 0.05; Table 2).

**Table 2:** Postoperative ROM in two groups (°)

**Group**	**6th week**	**3rd month**	**6th month**
Extension group (40)	87.62 ± 8.92	95.62 ± 6.51	110.87 ± 5.03
Flexion group (40)	98.95 ± 10.33	110.05 ± 4.93	115.05 ± 3.24
*t*	2.47	3.29	−0.06
*P*	0.03	0.04	0.72

### VAS pain score of the anterior knee and KSS

The VAS pain score of the anterior knee with 90° knee flexion in the flexion group was 2.02 ± 1.38 and 2.21 ± 0.87 at 6 weeks and 3 months postoperatively, whereas 2.57 ± 1.07 and 2.87 ± 0.83 in the extension group, respectively. A significant difference was observed between the two groups (*P* < 0.05), indicating that the closure in flexion can decrease postoperative anterior knee pain. However, no significant difference was observed between the two groups at 6 months postoperatively (*P* > 0.05; Table 3). Moreover, KSS was not significantly different between the flexion group and the extension group at 6 weeks, 3 months and 6 months postoperatively (*P* > 0.05; Table 4). This finding indicated that the location of wound closure has no effect on the postoperative joint function.

**Table 3:** Postoperative anterior knee VAS pain score in two groups

**Group**	**6th week**	**3rd month**	**6th month**
Extension group (40)	2.57 ± 1.07	2.87 ± 0.83	1.12 ± 0.68
Flexion group (40)	2.02 ± 1.38	2.21 ± 0.87	1.15 ± 0.73
*t*	−2.52	−2.69	−0.49
*P*	0.02	0.03	0.64

**Table 4:** Postoperative KSS in two groups

**Group**	**6th week**		**3rd month**		**6th month**	
	**Knee score**	**Function score**	**Knee score**	**Function score**	**Knee score**	**Function score**
Extension group (40)	48.76 ± 7.88	56.33 ± 6.29	89.23 ± 5.75	82.92 ± 9.57	90.56 ± 6.88	88.67 ± 7.85
Flexion group (40)	49.11 ± 10.39	58.96 ± 8.69	90.34 ± 5.85	85.63 ± 7.26	91.19 ± 6.31	92.46 ± 7.34
*t*	0.07	2.48	−0.77	0.64	2.11	715.5
*P*	0.92	0.81	0.43	0.57	0.37	0.67

### Postoperative complications

Knee extension lag was not observed in the two groups after surgery. An apparent complication of wound disunion, patella fracture and infection that required second surgery was not detected in the extension group and the flexion group.

## Discussion

Anterior knee pain after TKA is currently a major problem. Although most scholars consider anterior knee pain to be related to the pathogenic factor on patellofemoral joint, a clear consensus on the cause and treatment of anterior knee pain has not been achieved [12]. Previous studies reported that anterior knee pain is associated with different factors, including patient factors (pain threshold, preoperative activity and obesity), degree of patellar cartilage damage and wear, prosthesis factors (anatomy and non-anatomical design, rotating and stationary platform), surgery technique (extremely high joint line, patella thickness and height, patellar resurfacing and soft tissue treatment), postoperative pain management and rehabilitation exercises [2],[13]. In this study, preoperative ROM, VAS pain score of the anterior knee, KSS and other indexes are not significantly different between the two groups. All patients were treated with Smith-Nephew Genesis II prosthesis with patella forming but not with resurfacing or lateral retinacular release. The postoperative pain management and rehabilitation exercises in the two groups are the same, excluding the interference factors. After TKA, the effects of wound closure in flexion and extension on anterior knee pain are investigated.

High patellofemoral compartment pressure and pain with lateral retinaculum are the main factors for the anterior knee pain of patellofemoral osteoarthritis [14]. King et al. [7] suggested that anterior knee skin and soft tissue tension cause postoperative anterior knee pain and affect ROM recovery. Knee wound closure in extension can lead to relative shortening of the knee extension device and wound constraint. With the increase of knee flexion degree, the patellofemoral compartment pressure is elevated with pull and tearing of wound-wrinkled tissue, which then results in anterior knee discomfort. However, the knee wound closure in flexion can prevent this risk. The results of this study show that the VAS pain score of the anterior knee in flexion group is significantly lower than that in extension group at 6 weeks and 3 months postoperatively. The dominating nerve located in the lateral retinaculum can cause anterior knee pain. Thus, all patients in this study are not treated with lateral retinacular release. Therefore, the effect of lateral retinaculum on anterior knee pain can be prevented effectively.

The factors affecting ROM recovery after TKA mainly include patient selection, prosthesis design, surgical technique, postoperative rehabilitation, pain management and wound healing [15],[16]. Correct wound treatment can reduce tissue adhesion and promote ROM increase [16]. Improper wound treatment or infection may affect postoperative knee function recovery [17]. The tension of the wound soft tissue may increase in knee flexion for wound closure in extension, which results in risks of cracking and wound infection. However, wound closure in flexion can effectively promote ROM recovery after TKA [9]. The results in this study showed that ROM and ROM recovery in the flexion group are significantly higher and faster than those in the extension group, respectively, at 6 weeks and 3 months postoperatively. Masri et al. [10] assumed that wound closure in flexion is not beneficial for ROM recovery; however, the flexion angle in their study is only 60°, which is inadequate. In the current study, the flexion angle is 90°. Therefore, wound closure with flexion angle higher than 60° is effective. In situ alignment of soft tissue at incision is important for postoperative comfort and ROM, particularly for the precise alignment of articular capsule with quadriceps tendon. In this study, the incision of the articular capsule is marked using a suture (Figure 1B), which can tighten the sutured wound tissue in knee flexion and contribute to the precise alignment of wound. After wound closure, the knee is elevated in high flexion. Therefore, a high-tension pull on the soft-tissue wound with high tension results in the rebalancing of tension of sutured area and in the reduction of discomfort in flexion (Figure 1C). The ROM in the two groups is not significantly different at 6 months postoperatively. This finding indicates that wound closure in flexion is beneficial for early ROM recovery. Many factors affect function recovery after TKA. Wound closure is a soft-tissue treatment technique that should be used with other methods to promote knee function after TKA to obtain high ROM.

The effect of wound closure in flexion on knee extension lag is controversial. Previous research found that knee wound closure in extension can cause tissue accumulation, resulting in the relative shortening of the knee extension device. By contrast, wound closure in flexion does not harm the knee extension device and therefore does not cause knee extension lag [5]. In this study, knee extension lag is not observed in the two groups. However, preoperative quadriceps weakness is a reverse indication of wound closure in flexion [9]. Wound closure in flexion is beneficial to reduce intraoperative bleeding but may cause scratches on the surface of the femoral prosthesis. Thus, intraoperative protection is necessary. In flexion, the quadriceps tendon has close contact with soft tissues in the suprapatellar bursa. For wound closure in flexion, the quadriceps tendon may be sutured with the suprapatellar bursa tissue. The interspace between the quadriceps tendon and suprapatellar bursa should be checked, and the adhesion should be separated (Figure 1D). Otherwise, postoperative anterior knee pain in knee flexion will occur, which then affects ROM.

Many factors affect knee function recovery after TKA [18]. Wound closure is a treatment on soft tissue and has no decisive effect on postoperative knee function recovery. To increase postoperative knee flexion, wound closure can be used together with other methods that promote ROM recovery. Wound closure is a supplement to other technologies. In conclusion, marking the articular capsule incision, wound closure in flexion and high flexion after wound closure can effectively decrease anterior knee pain after TKA and promote the early recovery of ROM. We propose that wound closure can reduce anterior knee pain after TKA and can promote early recovery of ROM.

## Conclusions

The knee position during wound closure after TKA is not only critical but also very important for postoperative knee function recovery. Marking the articular capsule incision, wound closure in flexion and high flexion after wound closure can effectively decrease anterior knee pain after TKA and promote the early recovery of ROM. There is a significant difference between the two groups only in the early postoperative period, with no obvious difference upon follow-up of more than 6 months.

## Competing interests

All authors declare that they have no competing interests.

## Authors' contributions

SW and JX contributed substantially to the conception and design of the study. GH contributed in the acquisition of data. YW and JW analysed and interpreted the data. SW drafted the article and revised it critically for important intellectual content. All authors reviewed and edited the manuscript and approved the final version of the manuscript.
